# Genome Mining and Molecular Networking-Based Metabolomics of the Marine Facultative *Aspergillus* sp. MEXU 27854

**DOI:** 10.3390/molecules26175362

**Published:** 2021-09-03

**Authors:** Anahí Martínez-Cárdenas, Yuridia Cruz-Zamora, Carlos A. Fajardo-Hernández, Rodrigo Villanueva-Silva, Felipe Cruz-García, Huzefa A. Raja, Mario Figueroa

**Affiliations:** 1 Facultad de Química, Universidad Nacional Autónoma de México, Ciudad de México 04510, Mexico; amartinez@quimica.unam.mx (A.M.-C.); yuridiacruz@comunidad.unam.mx (Y.C.-Z.); carlosantoniofajardo@gmail.com (C.A.F.-H.); code.rodvil@gmail.com (R.V.-S.); fcg@unam.mx (F.C.-G.); 2 Department of Chemistry and Biochemistry, University of North Carolina at Greensboro, Greensboro, NC 27402, USA; haraja@uncg.edu

**Keywords:** marine-facultative fungi, genome mining, biosynthetic gene clusters, metabolomics, molecular networking

## Abstract

The marine-facultative *Aspergillus* sp. MEXU 27854, isolated from the Caleta Bay in Acapulco, Guerrero, Mexico, has provided an interesting diversity of secondary metabolites, including a series of rare dioxomorpholines, peptides, and butyrolactones. Here, we report on the genomic data, which consists of 11 contigs (N50~3.95 Mb) with a ~30.75 Mb total length of assembly. Genome annotation resulted in the prediction of 10,822 putative genes. Functional annotation was accomplished by BLAST searching protein sequences with different public databases. Of the predicted genes, 75% were assigned gene ontology terms. From the 67 BGCs identified, ~60% belong to the NRPS and NRPS-like classes. Putative BGCs for the dioxomorpholines and other metabolites were predicted by extensive genome mining. In addition, metabolomic molecular networking analysis allowed the annotation of all isolated compounds and revealed the biosynthetic potential of this fungus. This work represents the first report of whole-genome sequencing and annotation from a marine-facultative fungal strain isolated from Mexico.

## 1. Introduction

Fungi play an essential ecological role in both terrestrial and aquatic environments. It is now well-known that marine-derived fungi are an excellent source of novel natural products (NPs), from which numerous compounds have been isolated for drug development [1,2,3]. Marine *Aspergillus* have yielded over 30% of the total marine-microbial NPs [4]. This genus has 11 species listed in the World Register of Marine Species [5]. Among them, human pathogens and allergens (*A. fumigatus*), plant pathogens (*A. flavus*), model organisms (*A. nidulans*), and species with industrial applications (*A. niger* and *A. terreus*) can be found [5]. Efforts to sequence the genome of *Aspergillus* species began in 2005, allowing to answer questions related to evolution, ecological adaptation, and pathogenicity [6,7,8]. Since then, more robust and less expensive sequencing technologies have been developed, which has resulted in an increase in the number of *Aspergillus* genome assemblies in public databases. Currently, there are 374 released isolate assemblies in the NCBI Genbank (Table S1) [9]. From these, six are described as a complete genome, 19 at the chromosome level, and the rest at the scaffold or contig level (Table S1). *A. terreus*, the most important lovastatin producer, was the first member of the section *Terrei* whose genome was sequenced [7]; however, it is barely a scaffold. In our previous studies on the marine-facultative *Aspergillus* sp. MEXU 27854 (section *Terrei*), we isolated two new dioxomorpholines and three new derivatives, along with one new cyclic pentapeptide and the known compounds PF1233 A and B, and butyrolactone II [10,11]. Interestingly, these rare dioxomorpholines showed P-glycoprotein inhibition, which is associated with drug resistance in cancer therapy [10,12,13]. To date, the only partially known dioxomorpholines biosynthetic pathway was described for acu-dioxomorpholines A and B using a platform for screening and heterologous expression of intact and entire gene clusters that use fungal artificial chromosomes and metabolomic scoring (FAC-MS) [12]. Thus, studying the biosynthetic pathway of this group of compounds and its derivatives is of great interest. To better understand the biosynthesis of this class of compounds, we sequenced the genome of the strain MEXU 27854 and, using genome mining analysis, predicted a putative biosynthetic gene cluster (BGC) for the dioxomorpholines and other metabolites from this fungus.

## 2. Results and Discussion

### 2.1. General Genomic Features of Aspergillus sp. MEXU 27854

**Figure 1:**
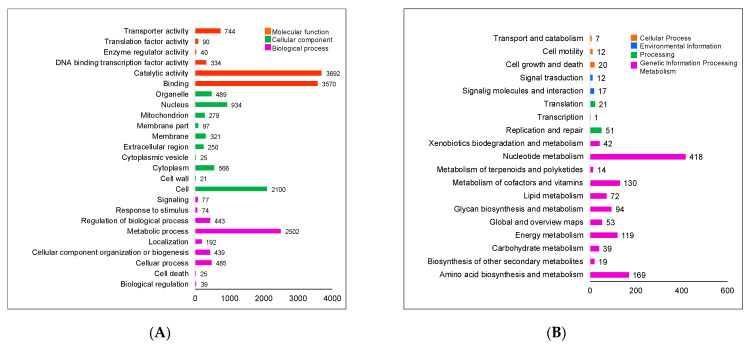
(**A**) GO and (**B**) KEGG functional annotations for *Aspergillus* sp. MEXU 27854.

The marine-facultative *Aspergillus* sp. MEXU 27854 was subjected to whole-genome sequencing using the PacBio technology. A total of 7 GB of sequencing data with an average read length of 9183 bp were generated. For assembly, reads over 12 kb in length were used (~84× coverage), which resulted in 11 contigs with a total and N50 read length of 30,756,112 bp and 3,946,678 bp, respectively (GenBank accession no. JAGMTT000000000). The sequencing quality statistics and predicted genomic information for this strain are shown in Table 1. The completeness of the assembly (10,822 total predicted genes; Table S2) was relatively high, as indicated by a BUSCO score of 99.5% (complete and single copy, 4153; duplicated, 15; fragmented, 6; missing, 14; n, 4191) when compared with genes conserved in the Eurotiales. Moreover, a total of 2744 predicted genes encoding hypothetical proteins without apparent homologs to currently available sequences were found in the fungal strain genome. According to the Gene Ontology (GO) database, 8078 predicted proteins that accounted for 75% of the entire genome were mainly distributed in four functional entries: binding, catalytic activity, transporter activity, and metabolic process (Figure 1A and Table S2). In addition, functional gene annotation was successfully assigned to 1116 (14%) putative proteins to their orthologs using the Kyoto Encyclopedia of Genes and Genomes (KEGG) database (Figure 1B and Table S3).

In an earlier study, MEXU 27854 was phylogenetically identified as belonging to the *Aspergillus* section *Terrei*, based on CaM phylogeny [14]. The strain occurred in an isolated position, in a clade sister to *A. niveus*, *A. carneus*, *A. alhabadii*, *A. neoindicus*, and *A. aureoterreus*. In the present study, sections of CaM and RPB2, single-copy protein-coding genes used for identification of *Aspergillus*, were extracted from the genome (Supplementary FASTA files). A BLAST search from these two regions also supported that MEXU 27845 showed homology with the above *Aspergillus* strains in the *Terrei* section, but only with ≥94% sequence similarity. These preliminary analyses suggest that MEXU 27845 might be a putatively new species of *Aspergillus* from Mexico.

**Figure 2:**
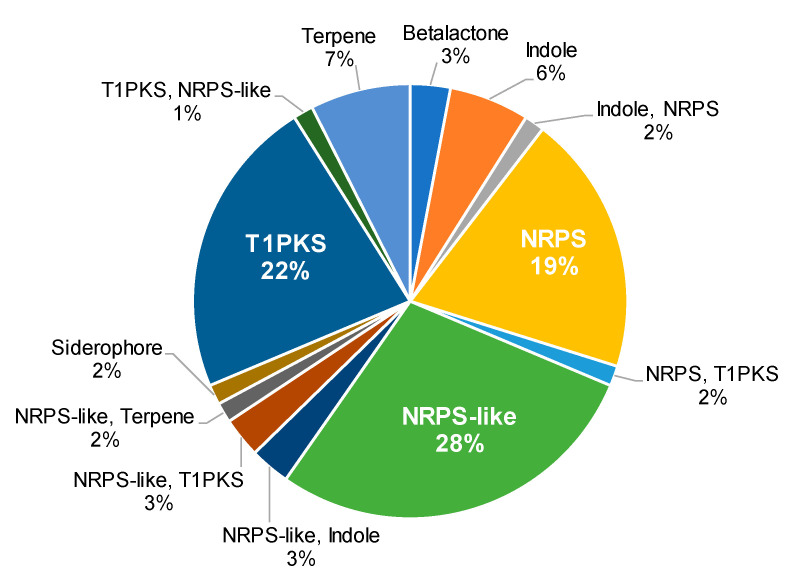
BGCs categories in *Aspergillus* sp. MEXU 27854.

AntiSMASH analysis of the *Aspergillus* sp. MEXU 27854 genome revealed the presence of 67 BGCs grouped in 13 categories (Figure 2). The non-ribosomal peptide synthases (NRPS) and NRPS-like proteins were the most abundant (>50%), followed by polyketide synthases (PKS) (~20%). NRPS are well-known multi-modular enzymes that catalyze the synthesis of highly diverse secondary metabolites in fungi and bacteria with varied activity, such as siderophores, pigments, immunosuppressants, antimicrobial, antiviral or anticancer agents, among others [15,16]. Further comparison of the 67 identified BGCs in the strain MEXU 27854 and the closely related *A. terreus* NIH2624 revealed the coexistence of 17 BGCs in both strains (Table 2). Clusters predicted by antiSMASH in MEXU 27854 but not in *A. terreus* NIH2624, were those associated with the production of fujikurins B-D (83% similarity; GenBank accession no. MZ503790) from *Fusarium fujikuroi* and *F. proliferatum* (Figure S1A) [17], and citrinin (56% similarity; GenBank accession no. MZ503795) from *A. niger*, *A. fumigatus*, *A. niveus*, among others (Figure S1B) [18]. In addition, burnettramic acid A and ochrindole A were also predicted by antiSMASH from *A. burnettii* (section *Flavi*) [19] and *A. steynii* (section *Circumdati*) [20], respectively (Table 2).

### 2.2. BGCs in Aspergillus sp. MEXU 27854

#### 2.2.1. Dioxomorpholines

**Figure 3:**
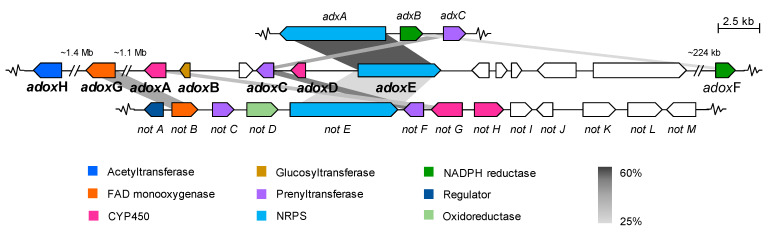
Synteny between predicted dioxomorpholines BGC (*adox*) from *Aspergillus* sp. MEXU 27854 and the contiguous homologous acu-dioxomorpholines BGC from *A. aculeatus* (GenBank accession no. KV878985.1) and notoamides BCG from *A. versicolor* NRRL35600 (GenBank accession no. JQ708194.1). Color-coded according to similarity functions of encoded enzymes between clusters. Grayscale bars linking proteins indicate amino acid identity (25% light gray, 60% dark gray).

In the *Aspergillus* sp. MEXU 27854 genome, contig 00 was predicted to contain seven BGCs, one of them involved in the dioxomorpholines biosynthesis (*adox* genes, GenBank accession no. MZ503791-MZ503794). Comparative analysis of *adox* with the acu-dioxomorpholines (*adx*) [12] and the indole-alkaloid notoamides (*not*) [21] biosynthetic genes (Table 3 and Table S4), predicted the uncharacterized dioxomorpholines BGC constituted for a NAD(P)-dependent reductase gene (*adoxF*), a NRPS gene (*adoxE*), a prenyltransferase gene (*adoxC*), and a CYP450 gene (*adoxA*), along with eight other genes (Figure 3). In addition, a FAD-dependent monooxygenase gene (*adoxG*) and a CoA-dependent acetyltransferase gene (*adoxH*), were proposed to participate in the final steps of the biosynthesis (Figure 3).

**Figure 4:**
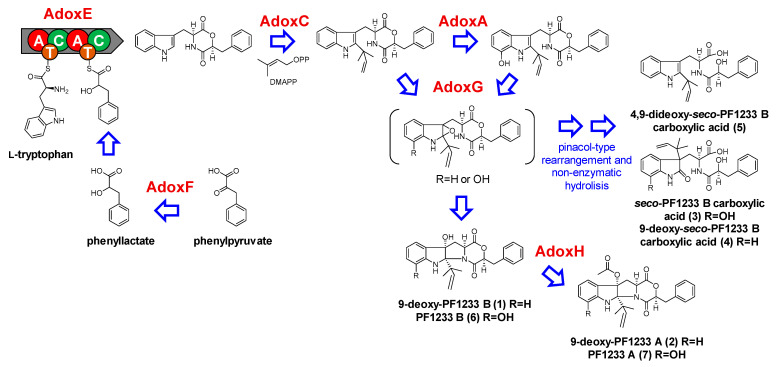
Proposed biosynthesis of dioxomorpholines (**1–7**) by *Aspergillus* sp. MEXU 27854.

Based on the structural similarity, gene function, and protein sequence resemblance between the dioxomorpholines and acu-dioxomorpholines [12], notoamides [21], and other indole alkaloids [22,23], we proposed reactions for the biosynthesis of the former in *Aspergillus* sp. MEXU 27854 (Figure 4 and Table S4). The first step is the phenylpyruvate reduction to phenyllactate by the NAD(P)-dependent reductase (AdoxF) (Figure S2). Then, a two-module NRPS (AdoxE) is responsible for the ester bond-forming condensation and assembly of the dioxomorpholine core from tryptophan and phenyllactate. The condensation domain of AdoxE was confirmed by comparison with *adxA* from *A. aculeatus* (50% sequence identity) [12], *notE* (24% sequence identity) [21], and several experimentally validated NRPS genes (Figure S3). Then, a reverse-prenylation at C2 of the indole from the AdoxE condensation product by AdoxC prenyltransferase (over 31% sequence similarity with several prenyltransferases), is likely to occur, similar to notoamides, paraherquamides, malbrancheamides, and brevianamides [21,24] (Figure S4). From there, a CYP450 monooxygenase AdoxA hydroxylates the indole at C8 of the dioxomorpholines, as the close homologue NotG (28% and 45% of identity and similarity) has been proposed to hydroxylate the C-H bond in deoxybrevianamide E (Figure S5) [21,24]. The next step involves an indole 2,3-epoxidation-initiated pinacol-like rearrangement catalyzed by AdoxG FAD monooxygenase, which exhibits 30% and 47% of identity and similarity with NotB (Figure S6), to produce 9-deoxy-PF1233 B (**1**), *seco*-PF1233 B carboxylic acid (**3**), 9-deoxy-*seco*-PF1233 B carboxylic acid (**4**), and PF1233 B (**6**). In addition, a non-enzymatic ring opening is expected to produce 4,9-dideoxy-*seco*-PF1233 B carboxylic acid (**5**). The final step in the dioxomorpholines biosynthesis is the acetylation of **1** and **6** to produce 9-deoxy-PF1233 A (**2**) and PF1233 A (**7**). As in several acetyl indole alkaloids, the acetylation step is catalyzed by a CoA-dependent acyltransferase [22,23]. The condensation domain of AdoxH was confirmed by comparison with different experimentally validated CoA-dependent acetyltransferases (45% and 65% of identity and similarity). This domain conserves the HXXXD and DFGWG motifs, essential for the catalytic activity of this family of enzymes (Figure S7).

#### 2.2.2. Cyclic Peptides

**Figure 5:**
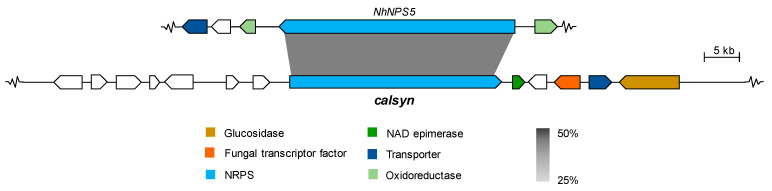
Synteny between predicted caletasin BGC (*calsyn*) from *Aspergillus* sp. MEXU 27854 and the contiguous homologous sansalvamides BGC from *F. solani* (accession no. XP_003044554.1). Color-coded according to similarity functions of encoded enzymes between clusters. Grayscale bars linking proteins indicate amino acid identity (25% light gray, 50% dark gray).

**Figure 6:**
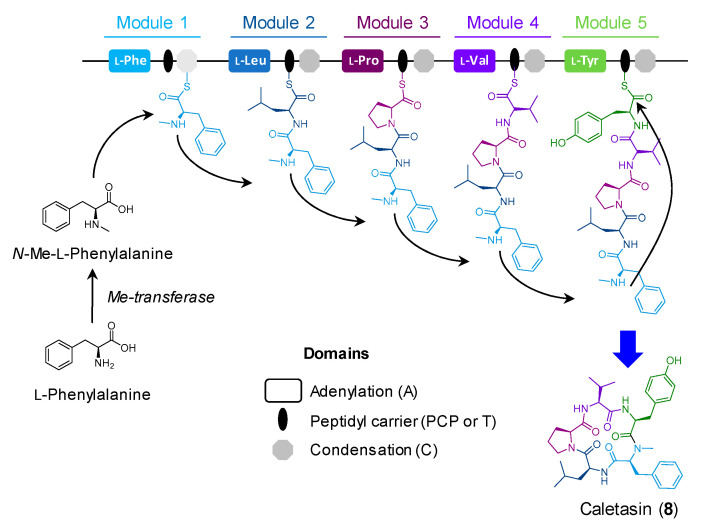
Proposed biosynthesis of caletasin (**8**) by *Aspergillus* sp. MEXU 27854.

Cyclic peptides from fungi are a well-known family of secondary metabolites with interesting structures and biological activities [25]. There are over 50 cyclic pentapeptides reported from fungi, and <20 produced by *Aspergillus* spp. [25]. Recently, we discovered the new *N*-methyl cyclic pentapeptide caletasin (**8**) from *Aspergillus* sp. MEXU 27854 [11], which is closely related to cotteslosins A and B produced by *A. versicolor* [26] and the sansalvamides produced by *F. solani* [27]. Genome mining, gene function, and protein sequence similarity analysis allowed the prediction of the caletasin BGC in the strain MEXU 27854 (Figure 5). The main protein, Calsyn, is a putative NRPS (GenBank accession no. MZ503796) organized in five essential adenylation and condensation domains for the pentapeptide formation (Figure 6). The *calsyn* NRPS gene sequence has 30% identity to the *NhNPS5* sansalvamide synthase (GenBank accession no. XP_003044554). Eight additional genes were also predicted without clear participation in the peptide biosynthesis.

#### 2.2.3. Butyrolactones

A chemical study of a fresh organic extract of *Aspergillus* sp. MEXU 27854 yielded 3-*O*-methylbutyrolactone II (**9**), which was identified by NMR and HRMS analysis (Figures S8 and S9, and Table S5) [28]. This secondary metabolite was isolated in 2015 from a gorgonian-derived *Aspergillus* strain [28] and is the methyl-derivative of butyrolactone II (**10**), previously identified in the MEXU 27854 strain [11]. Biosynthesis of **9** and **10** is carried out by an NRPS-like (*btyA*) and an S-adenosyl methionine (SAM)-methyltransferase (*btyB*), as demonstrated in *A. terreus* and *A. nidulans* [29,30,31]. As expected, the comparative analysis of the genomes of the strain MEXU 27854 and *A. terreus* showed high similarity (80% with *btyA* and 75% with *btyB*) in the BGC of these compounds (Table 4).

### 2.3. Mass-Spectrometry-Based Metabolomics Analysis

**Figure 7:**
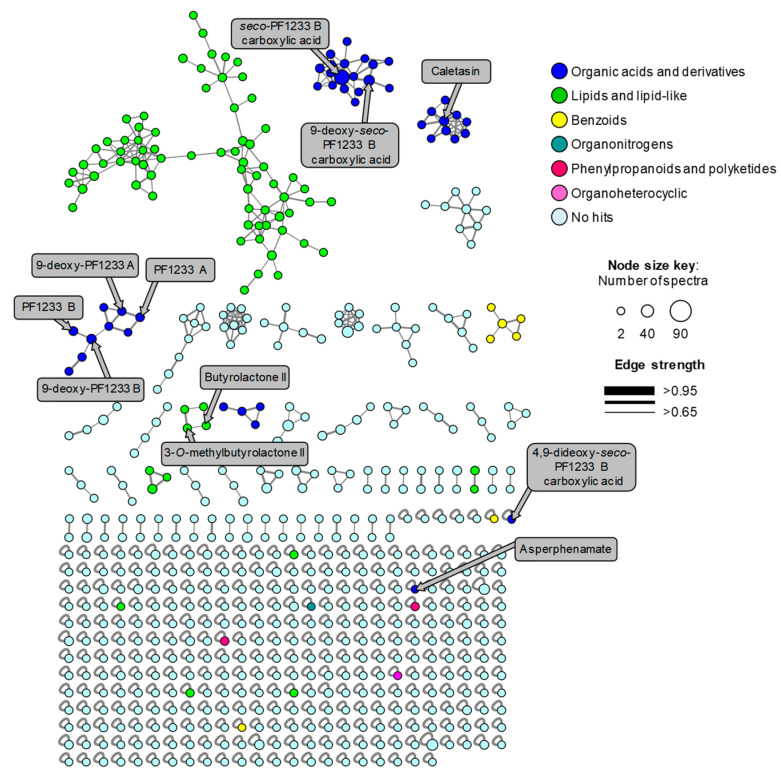
GNPS MN of *Aspergillus* sp. MEXU 27854 colored by super classes as indicated in the legend. Nodes represent parent ions. Edge strength show the chemical similarity between the MS/MS spectra. Compounds annotated manually and by GNPS are indicated in grey boxes with arrows pointing to the corresponding node (mass accuracy <5 ppm).

The most commonly used technique for targeted and untargeted metabolomic analysis of NPs is liquid chromatography-tandem mass spectrometry (LC-MS/MS). This platform provides high sensitivity and selectivity for compounds’ identification. Moreover, data analysis tools, such as principal component analysis and molecular networking (MN), are required to show the chemical space and diversity of the features or metabolites in the samples, which could be correlated to the functional phenotype of the natural source [32]. In this work, the Global Natural Products Social (GNPS) MN platform was used to further explore the biosynthetic potential of *Aspergillus* sp. MEXU 27854. The comprehensive network for this fungus was generated for spectra with a minimum of four fragment ions (Figure 7). Feature-based MN grouped the metabolite features into 52 chemical families (>3 nodes). Interestingly, only the known compounds asperphenamate and butyrolactone II (**10**) were annotated by GNPS (Table 5). Asperphenamate is produced by *A. flavus* and, even though it was not isolated, it is likely to be produced in strain MEXU 27854 because we found its BGC with a high percentage of similarity in the MEXU 27854 strain (Table 3). In addition, we were able to manually annotate all isolated compounds from this strain: dioxomorpholines and derivatives **1**–**7**, caletasin (**8**), and butyrolactones (**9** and **10**), because MS data from all pure compounds was included in the MN analysis (Table 5 and Figure 7).

## 3. Materials and Methods

### 3.1. Strain and DNA Isolation

*Aspergillus* sp. MEXU 27854 was isolated from sandy soil collected in the intertidal zone located in Caleta Bay, Acapulco, Guerrero, Mexico [10]. High-molecular-weight (HMW) genomic DNA was obtained from a pure culture of the strain using a modified phenol-chloroform DNA isolation protocol [33]. Briefly, the strain was cultivated on 30 mL of YESD (1% of yeast extract, 2% of soy peptone, 2% of dextrose) medium and incubated for 4 days at room temperature and 100 rpm (Lab Companion, Billerica, MA, USA). Ground mycelium (400–600 mg) was mixed with 300 µL of EB buffer (10 mM Tris-HCl, pH 8.5), then an equal volume of a PCI (phenol−chloroform−isoamyl alcohol) (25:24:1) solution was added. After vortexing for 1 min and centrifuging at 12,000 rpm for 5 min (HERMLE Labortechnik GmbH, Wehingen, Germany), the top aqueous layer was transferred into a new tube. An equal volume of a chloroform−isoamyl alcohol (24:1) solution was added to the new tube, vigorously vortexed for 1 min, and centrifuged (12,000 rpm) for 5 min. The aqueous layer was again transferred into a new tube. After cooling at −20 °C for 2 h with ethanol (100%) and NH_4_OAc (0.75 M final concentration), a precipitate was obtained. The pellet was washed with ethanol (70%) and air-dried for 2 min. RNAse A was used for DNA purification and re-extracted with PCI solution. HMW DNA was quantified using a UV-Vis BioDrop µLITE+ (Biochrom, Cambridge, United Kingdom). The quality of the genomic material was assessed on a Qubit 3.0 fluorometer (Thermo Fisher Scientific, Waltham, MA, USA) and an automated electrophoresis 4200 TapeStation system (Agilent Technologies, Santa Clara, CA, USA).

### 3.2. Sequencing and Assembly

Genome sequencing of the HMW DNA of the fungal strain was performed at the Centre d’expertise et de services Génome Québec in Quebec, Canada, using single-molecule real-time (SMRT) sequencing (0.5 SMRT cells from a single library) with the Sequel II system (Pacific Biosciences, Menlo Park, CA, USA). PacBio sequence data was error-corrected and assembled with the SMRT Link v9.0 software (Pacific Biosciences). Benchmarking Universal Single-Copy Orthologs (BUSCO) software was used to assess the completeness of genome assembly with single-copy orthologs [34]. BUSCO v2.0 was run on the genome assembly (using *A. terreus* genome as template). The lineage dataset of BUSCO was Eurotiales_odb10 (creation date: 2020-11-10).

### 3.3. Genome Annotation and BGC Prediction

Gene prediction was performed by AUGUSTUS version 3.3.3 [35]. The resulting gene sets were integrated to obtain the most comprehensive and non-redundant reference genes. The functional annotations of predicted genes were mainly based on homology to known annotated genes within different databases using the OmicsBox 1.4.12 platform as the main tool [36]. To achieve their corresponding annotation, protein models were aligned with the National Center for Biotechnology Information (NCBI) non-redundant (nr) database Blast2Go, InterPro [37], GO (http://geneontology.org/; accessed on 2 September 2021), and KEGG (https://www.genome.jp/kegg/; accessed on 2 September 2021). AntiSMASH fungal v.5.0 software was employed to predict the gene clusters of secondary metabolites with the cluster-finder algorithm for BGC border prediction and default settings [38]. Comparative bioinformatics analyses of the catalytic domains of the putative proteins and BGCs of the dioxomorpholines and other compounds were performed using ClustalX 2.1 [39].

### 3.4. Extract Preparation and Chemical Study

An organic extract (1.15 g) from a fresh solid culture (100 g of rice and 200 mL of H_2_O) of *Aspergillus* sp. MEXU 27854 was prepared as previously described [11]. From this, 1.0 g was fractionated via flash chromatography on a RediSep Rf Gold Si-gel column (40 g of Si-gel; Teledyne Inc., Thousand Oaks, CA, USA) using sequential mixtures of *n*-hexane−CHCl_3_−MeOH. Fifteen primary fractions were obtained according to their UV and ELSD profiles. Fraction eight (99 mg) was subjected to preparative HPLC (Kinetex C18 column 250 mm × 21.2 mm I.D., 5.0 μm, 100 Å; Phenomenex Inc., Torrance, CA, USA) using a gradient system from 60:40 to 100:0 of CH_3_CN-0.1% aqueous formic acid in 15 min at flow rate of 21.24 mL/min, yielding 3-*O*-methylbutyrolactone II (**9**) (40 mg, *t*_R_ = 5.2 min), which was characterized by comparing its NMR and HRMS spectra with those previously reported [28].

### 3.5. LC-MS/MS Analysis

A solution of the organic extract was prepared at 3 mg/mL and filtered with a 0.22 μm membrane before analyzing on an Acquity ultraperformance liquid chromatography (UPLC) system (Waters Corp., Milford, MA, USA) coupled to a Q Exactive Plus (Thermo Fisher Scientific, ThermoWaltham, MA, USA) mass spectrometer. An Acquity BEH C18 column was used for UPLC separations (50 mm × 2.1 mm I.D., 1.7 μm; Waters) with a flow rate of 0.3 mL/min equilibrated at 40 °C. The mobile phase consisted of a linear gradient between CH_3_CN-0.1% aqueous formic acid from 15% to 100% of CH_3_CN over 8 min, then held for 1.5 min at 100% CH3CN and returning to the starting conditions. High-resolution mass spectrometry (HRMS) data and MS/MS spectra were collected using an electrospray ionization (ESI) source (positive and negative modes) at a full scan range (*m*/*z* 150−2000), with the following settings: capillary voltage, 5 V; capillary temperature, 300 °C; tube lens offset, 35 V; spray voltage, 3.80 kV; sheath and auxiliary gas flow, 30 and 20 arbitrary units.

### 3.6. Metabolomic Analysis

Raw MS/MS data from samples (extract and pure compounds **1**–**10**), solvents (blank), and culture media (blank) were converted to .mzML file format using the Global Natural Products Social (GNPS) quick start converter and uploaded to the GNPS server (http://gnps.ucsd.edu; accessed on 2 September 2021) [40]. MN was performed using the reference GNPS data analysis workflow [40]. Briefly, for spectral networks, a parent mass and fragment ion tolerance of 0.01 and 0.02 Da were considered. Different parameters (cosine and minimum matched fragment ions) were evaluated to determine the best networking conditions. For edges construction, a cosine score over 0.70 was fitted. A minimum of four matching ions, two nodes at least in the top 10 cosine scores, and 100 maximum connected components were considered for the analysis. Afterwards, the network spectra were searched against GNPS spectral libraries, considering scores above 0.70 and at least four matched ions. The chemical classification was carried out with the MolNetEnhancer GNPS tool, where the score is calculated representing what percentage of nodes within a molecular family are attributed to a given chemical class [41]. GNPS spectral libraries and graphic visualization of the MN were performed in Cytoscape 3.8.1. [42]. Manually dereplication was assessed using UV-absorption maxima and HRMS-MS/MS data against MS/MS data of **1**–**10**. The annotation of these compounds was at confidence level 2 according to the metabolomics standards initiative [43] and exact mass accuracy <5 ppm.

### 3.7. Data Availability

The Whole Genome Shotgun (WGS) project of *Aspergillus* sp. MEXU 27,854 has been deposited at DDBJ/ENA/GenBank under the accession JAGMTT000000000 (BioSample SAMN17220881). The version described in this paper is version JAGMTT010000000. LC-MS/MS data can be accessed at MassIVE (accession no. MSV000086851). MN can be accessed at http://gnps.ucsd.edu/ProteoSAFe/status.jsp?task=4167727356f344998169f9a3ebd55fc9 (full data; accessed on 2 September 2021) and http://gnps.ucsd.edu/ProteoSAFe/status.jsp?task=414612a368b644ab9b9ac4fddf2e7a20 (MolNetEnhancer analysis; accessed on 2 September 2021).

## 4. Conclusions

This work represents the first report of genome sequencing and annotation of a marine-facultative fungal strain isolated from Mexico. Genomic data analysis and secondary metabolites biosynthetic potential of this fungus was assessed by the prediction of over 10,000 putative genes and 67 BGCs. Our work provides additional insight into the biosynthetic pathway of dioxomorpholines, whose BGC is only partially known. Finally, metabolomic MN analysis allowed us to highlight the biosynthetic capability of the fungus and to contribute to the GNPS community by providing data of rare compounds.

## Figures and Tables

**Table 1:** Genome summary statistics for *Aspergillus* sp. MEXU 27854.

**Contig Characteristics**	
Total number	11
Total length (bp)	30,756,112
N_50_	3,946,678
L_50_	4
Max. length (bp)	5,241,077
GC (%)	53
**Genome Characteristics**	
Genome assembly (MB)	30.75
Predicted protein coding sequences	10,822
% GO terms	75
Average gene length (bp)	2934

**Table 2:** Clusters prediction similarity between *Aspergillus* sp. MEXU 27854 and *A. terreus* NIH2624.

Cluster	Contig/Location/Similarity (%)					
	*Aspergillus* sp. MEXU 27854			*A. terreus* NIH2624		
Asperfuranone	4	2,673,561–2,721,442	18	11	634,985–694,556	81
Asperphenamate	3	271,678–331,344	75	14	139,864–204,269	100
Azanigerone A	8	1,592,427–1,640,144	20	5	1,778,394–1,863,542	20
Burnettramic acid A	3	390,502–438,846	22	14	209,359–297,831	22
Citreoviridin	1	862,457–908,022	80	15	353,636–401,160	100
Clavaric acid	6	1,529,334–1,551,729	100	12	102,884–121,870	100
Clavaric acid	6	1,529,334–1,551,729	100	12	102,884–121,870	100
Dimethylcoprogen	4	2,608,491–2,654,299	100	11	195,335–278,566	100
Dimethylcoprogen	4	2,608,491–2,654,299	100	11	195,335–278,566	100
Duclauxin	5	102,877–149,557	21	11	195,335–278,566	14
Monascorubrin	4	2,673,561–2,721,442	100	4	1,778,394–1,863,542	100
Naphthopyrone	4	2,500,933–2,543,731	100	8	1,584,471–1,627,478	100
Nidulanin A	6	2,333,314–2,390,373	50	1	620,230–676,423	100
Ochrindole A	6	3,324,939–3,362,374	29	1	1,948,860–1,985,238	11
Pyranonigrin E	6	2,504,128–2,546,577	100	1	786,879–829,500	100
Squalestatin S1	8	1,544,948–1,587,995	60	12	501,832–541,227	60
Terrequinone A	6	3,324,939–3,362,374	100	1	1,948,860–1,985,238	60

**Table 3:** Comparative analysis of BGCs of dioxomorpholines (*adox*), acu-dioxomorpholines (*adx*), and notoamides (*not*).

Adox Proteins (AA)	Function (% Identity to Corresponding Adx/Not Proteins) ^a^	Adx Proteins (AA)	Function (% Identity to Corresponding Not Proteins) ^a^
g7012/AdoxG	FAD monooxygenase	adxA	NRPS [A-T-C-A-T-C]
(426)	(30%, NotB)	(2413)	(24%, NotE)
g7440/AdoxA	CYP450 monooxygenase	adxB	Reductase
(490)	(28%, NotG)	(378)	(−)
g7441/AdoxB	Glucosyltransferase	adxC	Prenyltransferase
(155)	(−)	(422)	(35%, NoF)
g7442	CYP450 monooxygenase		
(261)	(−)		
g7443/AdoxC	Prenyltransferase		
(385)	(32%, AdxC/43%, NotF)		
g7444/AdoxD	CYP450 monooxygenase		
(248)	(−)		
g7445/AdoxE	NRPS [A-T-C-A-T-C]		
(2008)	(50%, AdxA/24%, NotE)		
g7446	Unknown		
(455)	(−)		
g7447	Unknown		
(252)	(−)		
g7448	Unknown		
(191)	(−)		
g7449	Unknown		
(915)	(−)		
g7450	Transcriptional factor		
(2343)	(−)		
g7531/AdoxF	NADPH-Reductase		
(295)	(22%, AdxB)		
g2501/AdoxH	Acetyltransferase		
(531)	(−)		

^a^ Gene function predicted using BLAST search. (−), homology cannot be calculated due to unrelatedness.

**Table 4:** Comparative analysis of the butyrolactone genes of *Aspergillus* sp. MEXU 27854.

Proteins (AA)	GenBank Accession No.	Closest GenBank Homolog	Amino Acid Identity (%)
g572 (930)	MZ503789	ATEG_02815 Non-ribosomal peptide synthetase *btyA* Butyrolactone IIa synthetase (*A. terreus* NIH2624) Q0CU19.2	746/931 (80%)
		Putative non-ribosomal peptide synthetase (*Cladonia uncialis* subsp. *Uncialis*) ANM86632.1	431/935 (46%)
		Non-ribosomal peptide synthetase (*A. tanneri*) XP_033423325.1	406/934 (43%)
g573 (271)		ATEG_02816 Methyltransferase *btyB* (*A. terreus* NIH2624) XP_001211994.1	203/269 (75%)
		Hypothetical protein ATETN484_0005003700 (*A. terreus*) GES60578.1	223/271 (82%)

**Table 5:** Chemical annotation by GNPS and by comparison with previously isolated compounds from *Aspergillus* sp. MEXU 27854 in the MN.

Compound	Observed Ion ^a^	Adduct	Molecular Formula	Exact Mass ^c^	Mass Accuracy (ppm)
9-deoxy-PF1233 B (**1**)	419.195	[M + H]^+^	C_25_H_26_N_2_O_4_	418.1893	−3.7
9-deoxy-PF1233 A (**2**)	461.206	[M + H]^+^	C_27_H_28_N_2_O_5_	460.1998	−2.4
*seco*-PF1233 B carboxylic acid (**3**)	453.202	[M + H]^+^	C_25_H_28_N_2_O_6_	452.1947	0.0
9-deoxy-*seco*-PF1233 B carboxylic acid (**4**)	437.206	[M + H]^+^	C_25_H_28_N_2_O_5_	436.1998	−2.5
4,9-dideoxy-*seco*-PF1233 B carboxylic acid (**5**)	421.211	[M + H]^+^	C_25_H_28_N_2_O_4_	420.2049	−2.4
PF1233 B (**6**)	435.191	[M + H]^+^	C_25_H_26_N_2_O_5_	434.1842	−1.0
PF1233 A (**7**)	477.202	[M + H]^+^	C_27_H_28_N_2_O_6_	476.1941	0.0
Caletasin (**8**)	634.359	[M + H]^+^	C_35_H_47_N_5_O_6_	633.3526	−1.4
3-*O*-methylbutyrolactone II (**9**)	371.112	[M + H]^+^	C_20_H_18_O_7_	370.1052	+2.7
Butyrolactone II (**10**)	357.097	[M + H]^+^	C_19_H_16_O_7_	356.0896	+2.7
Asperphenamate ^b^	507.228	[M + H]^+^	C_32_H_30_N_2_O_4_	506.2206	+2.0

^a^ Values taken from GNPS analysis; ^b^ annotated by GNPS; ^c^ HRMS data from each isolated compound.

## Data Availability

The authors confirm that the data supporting the findings of this study are available within the article and its supplementary material.

## Supplementary Materials

The following are available online, Table S1. Data of *Aspergillus* spp. genomes in the NCBI; Table S2. Total predicted genes in the *Aspergillus* sp. MEXU 27854 genome and GO terms; Table S3. KEGG pathway classification in the *Aspergillus* sp. MEXU 27854 genome; Table S4. Deduced functions of open reading frames (ORFs) in the *adox* BGC; Table S5. ^1^H NMR data of 3-*O*-methylbutyrolactone II (9); Figure S1. (A) Fujikurins BGC from *F. fujikuroi* IMI 58289 (*fujA*) and predicted for *Aspergillus* sp. MEXU 27854. (B) Citrinin BGC from *M. ruber* (*citH*) and predicted for *Aspergillus* sp. MEXU 27854; Figure S2. Clustal analysis of the putative g7531 phenylpyruvate reductase (AdoxF); Figure S3. Clustal analysis of putative g7445 NPRS (AdoxE); Figure S4. Clustal analysis of putative g7443 aromatic prenyltransferase (AdoxC); Figure S5. Clustal analysis of the putative g7440 cytochrome P450 monooxygenase (AdoxA); Figure S6. Clustal analysis of the putative g7012 FAD monooxygenase (AdoxG); Figure S7. Clustal analysis of the putative g2501 CoA acetyltransferase (AdoxH); Figure S8. ^1^H NMR spectrum of 3-*O*-methylbutyrolactone II (9); Figure S9. UV and HRESIMS spectra of 3-*O*-methylbutyrolactone II (9). FASTA files of CaM and RPB2 single-copy protein-coding gene sequences.

## Sample Availability

Samples of the compounds **1**–**10** are available from the corresponding author.

## References

[1] Hagestad O.C., Andersen J.H., Altermark B., Hansen E., Rämä T. (2020). Cultivable marine fungi from the arctic archipelago of Svalbard and their antibacterial activity. Mycology.

[2] Hu J., Li Z., Gao J., He H., Dai H., Xia X., Liu C., Zhang L., Song F. (2019). New diketopiperazines from a marine-derived fungus strain *Aspergillus versicolor* MF180151. Mar. Drugs.

[3] Nagano Y., Miura T., Tsubouchi T., Lima A.O., Kawato M., Fujiwara Y., Fujikura K. (2020). Cryptic fungal diversity revealed in deep-sea sediments associated with whale-fall chemosynthetic ecosystems. Mycology.

[4] Zhao C., Liu H., Zhu W. (2016). New natural products from the marine-derived Aspergillus fungi—A review. Acta Microbiol. Sin..

[5] Horton T., Kroh A., Ahyong S., Bailly N., Boyko C.B., Brandão S.N., Gofas S., Hooper J.N.A., Hernandez F., Holovachov O. World Register of Marine Species (WoRMS). https://www.marinespecies.org.

[6] Galagan J.E., Calvo S.E., Cuomo C., Ma L.J., Wortman J.R., Batzoglou S., Lee S.I., Baştürkmen M., Spevak C.C., Clutterbuck J. (2005). Sequencing of *Aspergillus nidulans* and comparative analysis with *A. fumigatus* and *A. oryzae*. Nature.

[7] Machida M., Asai K., Sano M., Tanaka T., Kumagai T., Terai G., Kusumoto K., Arima T., Akita O., Kashiwagi Y. (2005). Genome sequencing and analysis of *Aspergillus oryzae*. Nature.

[8] Nierman W.C., Pain A., Anderson M.J., Wortman J.R., Kim H.S., Arroyo J., Berriman M., Abe K., Archer D.B., Bermejo C. (2005). Genomic sequence of the pathogenic and allergenic filamentous fungus *Aspergillus fumigatus*. Nature.

[9] Coordinators N.R. (2016). Database resources of the national center for biotechnology information. Nucleic Acids Res..

[10] Aparicio-Cuevas M.A., Rivero-Cruz I., Sánchez-Castellanos M., Menéndez D., Raja H.A., Joseph-Nathan P., González M.D.C., Figueroa M. (2017). Dioxomorpholines and derivatives from a marine-facultative *Aspergillus* species. J. Nat. Prod..

[11] Aparicio-Cuevas M.A., González M.D.C., Raja H., Rivero-Cruz I., Kurina S.J., Burdette J.E., Oberlies N.H., Figueroa M. (2019). Metabolites from the marine-facultative *Aspergillus* sp. MEXU 27854 and *Gymnoascus hyalinosporus* MEXU 29901 from Caleta Bay, Mexico. Tetrahedron Lett..

[12] Robey M.T., Ye R., Bok J.W., Clevenger K.D., Islam M.N., Chen C., Gupta R., Swyers M., Wu E., Gao P. (2018). Identification of the first diketomorpholine biosynthetic pathway using FAC-MS technology. ACS Chem. Biol..

[13] Khalil Z.G., Huang X.C., Raju R., Piggott A.M., Capon R.J. (2014). Shornephine A: Structure, chemical stability, and P-glycoprotein inhibitory properties of a rare diketomorpholine from an australian marine-derived *Aspergillus* sp.. J. Org. Chem..

[14] Samson R.A., Peterson S.W., Frisvad J.C., Varga J. (2011). New species in *Aspergillus* section *Terrei*. Stud. Mycol..

[15] Le Govic Y., Papon N., Le Gal S., Bouchara J.P., Vandeputte P. (2019). Non-ribosomal peptide synthetase gene clusters in the human pathogenic fungus *Scedosporium apiospermum*. Front. Microbiol..

[16] Martínez-Núñez M.A., López V.E.L. (2016). Nonribosomal peptides synthetases and their applications in industry. Sustain. Chem. Process..

[17] Von Bargen K.W., Niehaus E.M., Krug I., Bergander K., Würthwein E.U., Tudzynski B., Humpf H.U. (2015). Isolation and structure elucidation of fujikurins A-D: Products of the PKS19 gene cluster in *Fusarium fujikuroi*. J. Nat Prod..

[18] Hajjaj H., Klaebe A., Loret M.O., Goma G., Blanc P.J., Francois J. (1999). Biosynthetic pathway of citrinin in the filamentous fungus *Monascus ruber* as revealed by ^13^C nuclear magnetic resonance. Appl. Environ. Microbiol..

[19] Li H., Gilchrist C.L.M., Lacey H.J., Crombie A., Vuong D., Pitt J.I., Lacey E., Chooi Y.H., Piggott A.M. (2019). Discovery and heterologous biosynthesis of the burnettramic acids: Rare PKS-NRPS-derived bolaamphiphilic pyrrolizidinediones from an australian fungus, *Aspergillus burnettii*. Org. Lett..

[20] Kjærbølling I., Vesth T.C., Frisvad J.C., Nybo J.L., Theobald S., Kuo A., Bowyer P., Matsuda Y., Mondo S., Lyhne E.K. (2018). Linking secondary metabolites to gene clusters through genome sequencing of six diverse *Aspergillus* species. Proc. Natl. Acad. Sci. USA..

[21] Li S., Srinivasan K., Tran H., Yu F., Finefield J.M., Sunderhaus J.D., McAfoos T.J., Tsukamoto S., Williams R.M., Sherman D.H. (2012). Comparative analysis of the biosynthetic systems for fungal bicyclo [2.2.2] diazaoctane indole alkaloids: The (+)/(−)-notoamide, paraherquamide and malbrancheamide pathways. Med. Chem. Commun..

[22] Ahmad B., Banerjee A., Tiwari H., Jana S., Bose S., Chakrabarti S. (2018). Structural and functional characterization of the vindoline biosynthesis pathway enzymes of *Catharanthus roseus*. J. Mol. Model..

[23] Ma X., Koepke J., Panjikar S., Fritzsch G., Stöckigt J. (2005). Crystal structure of vinorine synthase, the first representative of the BAHD superfamily. J. Biol. Chem..

[24] Ye Y., Du L., Zhang X., Newmister S.A., McCauley M., Alegre-Requena J.V., Zhang W., Mu S., Minami A., Fraley A.E. (2020). Fungal-derived brevianamide assembly by a stereoselective semipinacolase. Nat. Catal..

[25] Wang X., Lin M., Xu D., Lai D., Zhou L. (2017). Structural diversity and biological activities of fungal cyclic peptides, excluding cyclodipeptides. Molecules.

[26] Fremlin L.J., Piggott A.M., Lacey E., Capon R.J. (2009). Cottoquinazoline A and cotteslosins A and B, metabolites from an australian marine-derived strain of *Aspergillus versicolor*. J. Nat Prod..

[27] Romans-Fuertes P., Sondergaard T.E., Sandmann M.I.H., Wollenberg R.D., Nielsen K.F., Hansen F.T., Giese H., Brodersen D.E., Sørensen J.L. (2016). Identification of the non-ribosomal peptide synthetase responsible for biosynthesis of the potential anti-cancer drug sansalvamide in *Fusarium solani*. Curr. Genet..

[28] Chen M., Wang K.-L., Liu M., She Z.-G., Wang C.-Y. (2015). Bioactive steroid derivatives and butyrolactone derivatives from a Gorgonian-derived *Aspergillus* sp. fungus. Chem. Biodivers..

[29] Guo C.J., Knox B.P., Sanchez J.F., Chiang Y.M., Bruno K.S., Wang C.C. (2013). Application of an efficient gene targeting system linking secondary metabolites to their biosynthetic genes in *Aspergillus terreus*. Org. Lett..

[30] Guo C.J., Sun W.W., Bruno K.S., Oakley B.R., Keller N.P., Wang C.C.C. (2015). Spatial regulation of a common precursor from two distinct genes generates metabolite diversity. Chem. Sci..

[31] van Dijk J.W.A., Wang C.C.C. (2018). Expanding the chemical space of nonribosomal peptide synthetase-like enzymes by domain and tailoring enzyme recombination. Org. Lett..

[32] Aron A.T., Gentry E.C., McPhail K.L., Nothias L.F., Nothias-Esposito M., Bouslimani A., Petras D., Gauglitz J.M., Sikora N., Vargas F. (2020). Reproducible molecular networking of untargeted mass spectrometry data using GNPS. Nat. Protoc..

[33] Kumar M., Mugunthan M. (2018). Evaluation of three DNA extraction methods from fungal cultures. Med. J. Armed Forces India.

[34] Simão F.A., Waterhouse R.M., Ioannidis P., Kriventseva E.V., Zdobnov E.M. (2015). BUSCO: Assessing genome assembly and annotation completeness with single-copy orthologs. Bioinformatics.

[35] Stanke M., Diekhans M., Baertsch R., Haussler D. (2008). Using native and syntenically mapped cDNA alignments to improve de novo gene finding. Bioinformatics.

[36] Götz S., García-Gómez J.M., Terol J., Williams T.D., Nagaraj S.H., Nueda M.J., Robles M., Talón M., Dopazo J., Conesa A. (2008). High-throughput functional annotation and data mining with the Blast2GO suite. Nucleic Acids Res..

[37] Blum M., Chang H.-Y., Chuguransky S., Grego T., Kandasaamy S., Mitchell A., Nuka G., Paysan-Lafosse T., Qureshi M., Raj S. (2021). The InterPro protein families and domains database: 20 years on. Nucleic Acids Res..

[38] Blin K., Shaw S., Steinke K., Villebro R., Ziemert N., Lee S.Y., Medema M.H., Weber T. (2019). AntiSMASH 5.0: Updates to the secondary metabolite genome mining pipeline. Nucleic Acids Res..

[39] Larkin M.A., Blackshields G., Brown N.P., Chenna R., McGettigan P.A., McWilliam H., Valentin F., Wallace I.M., Wilm A., Lopez R. (2007). Clustal W and Clustal X version 2.0. Bioinformatics.

[40] Wang M., Carver J.J., Phelan V.V., Sanchez L.M., Garg N., Peng Y., Nguyen D.D., Watrous J., Kapono C.A., Luzzatto-Knaan T. (2016). Sharing and community curation of mass spectrometry data with global natural products social molecular networking. Nat. Biotechnol..

[41] Ernst M., Kang K.B., Caraballo-Rodríguez A.M., Nothias L.F., Wandy J., Chen C., Wang M., Rogers S., Medema M.H., Dorrestein P.C. (2019). MolNetEnhancer: Enhanced molecular networks by integrating metabolome mining and annotation tools. Metabolites.

[42] Shannon P., Markiel A., Ozier O., Baliga N.S., Wang J.T., Ramage D., Amin N., Schwikowski B., Ideker T. (2003). Cytoscape: A software environment for integrated models of biomolecular interaction networks. Genome Res..

[43] Marquès M., Mari M., Audí-Miró C., Sierra J., Soler A., Nadal M., Domingo J.L. (2016). Climate change impact on the PAH photodegradation in soils: Characterization and metabolites identification. Environ. Int..

