# Supplementary material for: Genome Mining and Molecular Networking-Based Metabolomics of the Marine Facultative Aspergillus sp. MEXU 27854
Source: Molecules. 2021 Sep 3;26(17):5362. doi: 10.3390/molecules26175362 (PMC8433890; doi:10.3390/molecules26175362)
Supplement: Supplementary file 1 [file molecules-26-05362-s001.zip › SI Figures_Aspergillus sp MEXU 27854_Revised.pdf]

SUPPORTING INFORMATION FIGURES

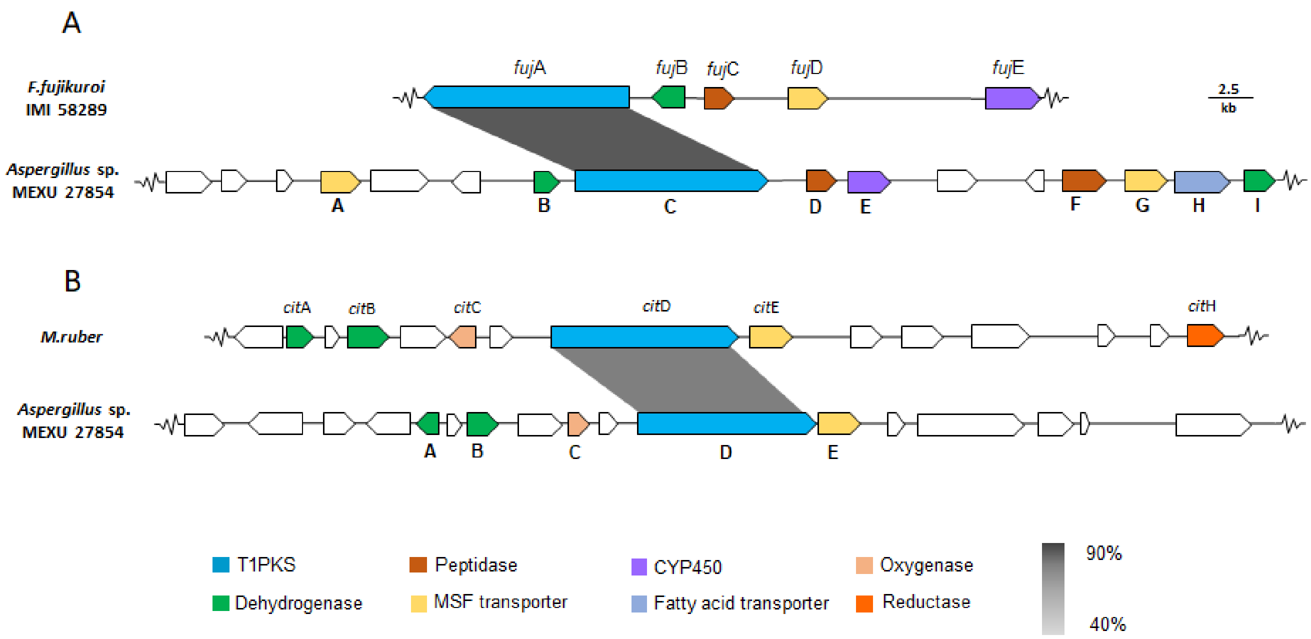

Figure S1. (**A**) Fujikurins BGC from *F. fujikuroi* IMI 58289 (*fujA*; GenBank accession no. HF679030.1.) and predicted for *Aspergillus* sp. MEXU 27854 (GenBank accession no. MZ503790). (**B**) Citrinin BGC from *M. ruber* (*citH*; GenBank accession no. KT781075.1) and predicted for *Aspergillus* sp. MEXU 27854 (GenBank accession no. MZ503795). Gene abbreviations: type I Polyketide synthase (T1PKS), major facilitator superfamily transporter (MFS transporter). Domain abbreviations: ketoreductase (KR), starter acyltransferase (SAT), ketosynthase (KS), acyltransferase (AT), peptidyl carrier protein (P), *C*-methyltransferase (cMT), thioesterase (TE), and dehydratase (DH). Color-coded according to similarity functions of encoded enzymes between clusters.

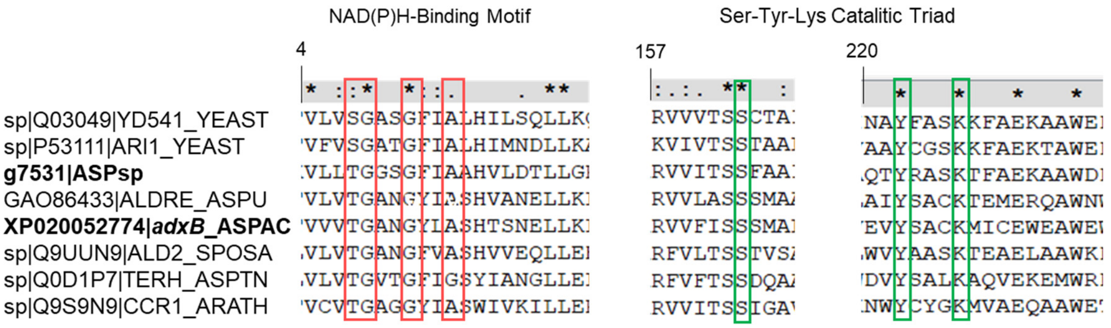

Figure S2. Clustal analysis of the putative g7531 phenylpyruvate reductase (AdoxF). AdoxF contains an NAD(P)H binding motif (red box) and a Ser-Tyr-Lys catalytic triad (green box) as a short chain dehydrogenase family of enzymes. The sequence numbering is shown according to AdoxF.

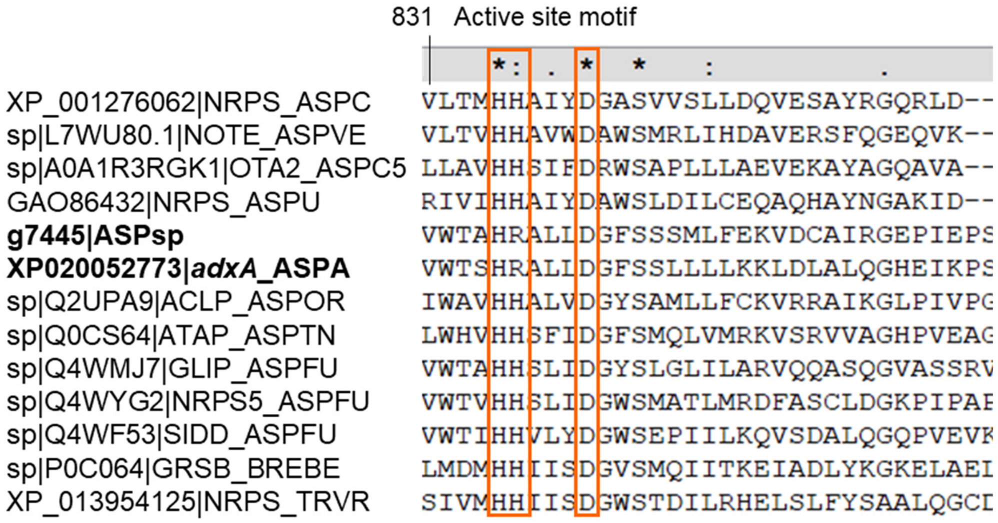

Figure S3. Clustal analysis of putative g7445 NPRS (AdoxE). AdoxE contains a condensation domain (orange boxes) as the AdxA homologous and other synthases experimentally characterized, and an arginine in place on the catalytic histidine as observed in several bacterial and fungal condensation domains. The sequence numbering is shown according to AdoxE.

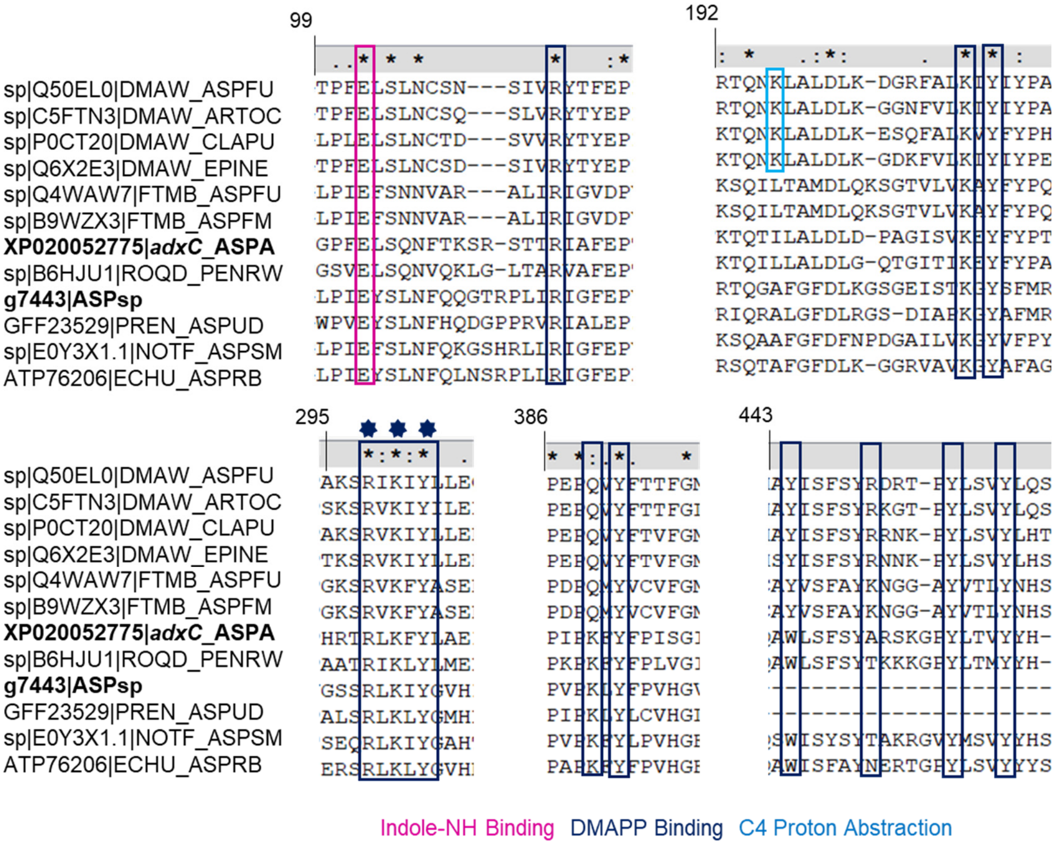

Figure S4. Clustal analysis of putative g7443 aromatic prenyltransferase (AdoxC). AdoxC contains a glutamate predicted to bind to the substrate indole nitrogen (pink box) and residues predicted to bind to DMAPP (dark blue boxes). In addition, AdoxC lacks a lysine (light blue box), similar to AdxC and NotF, which is proposed to remove the proton from the C4 position in 4-dimethylallyl tryptophan synthases, which could be due to the possible prenylation at C3. The sequence numbering is shown according to AdoxC.

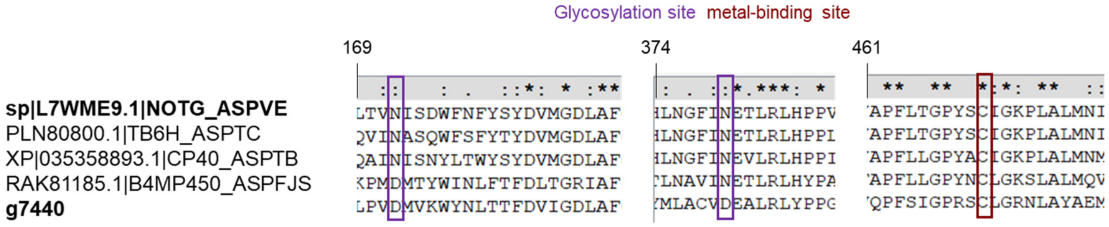

Figure S5. Clustal analysis of the putative g7440 cytochrome P450 monooxygenase (AdoxA). AdoxA contains an aspartic acid instead of asparagine in the glycosylation site (purple boxes). Similar to NotG, AdoxA contains a cysteine in the metal-binding site (red box). The sequence numbering is shown according to AdoxA.

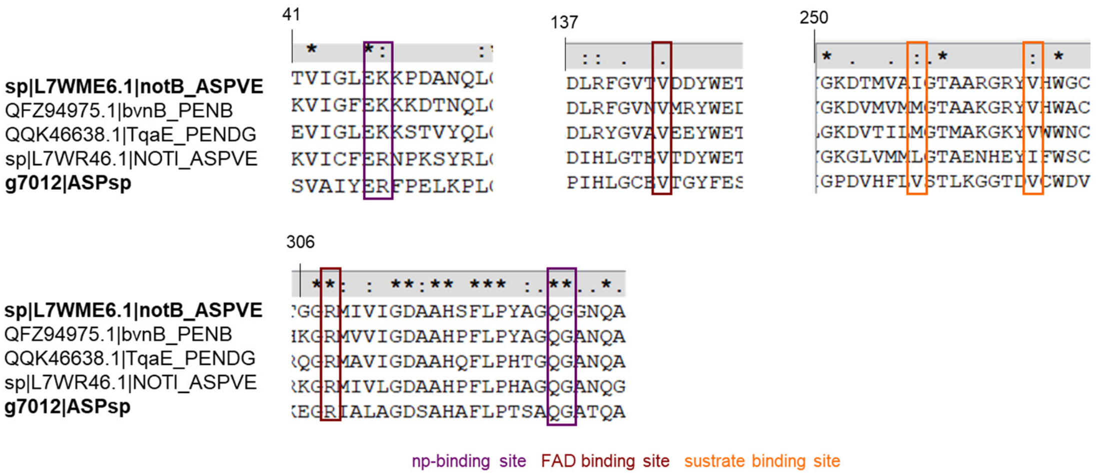

Figure S6. Clustal analysis of the putative g7012 FAD-monooxygenase (AdoxG). AdoxG contains two FAD (red boxes), and nucleoprotein binding motif (purple boxes) similar to other indole alkaloid monooxygenases. The substrate binding site (orange boxes) in AdoxG contains two valines instead of an isoleucine and a valine as in NotB. The sequence numbering is shown according to AdoxG.

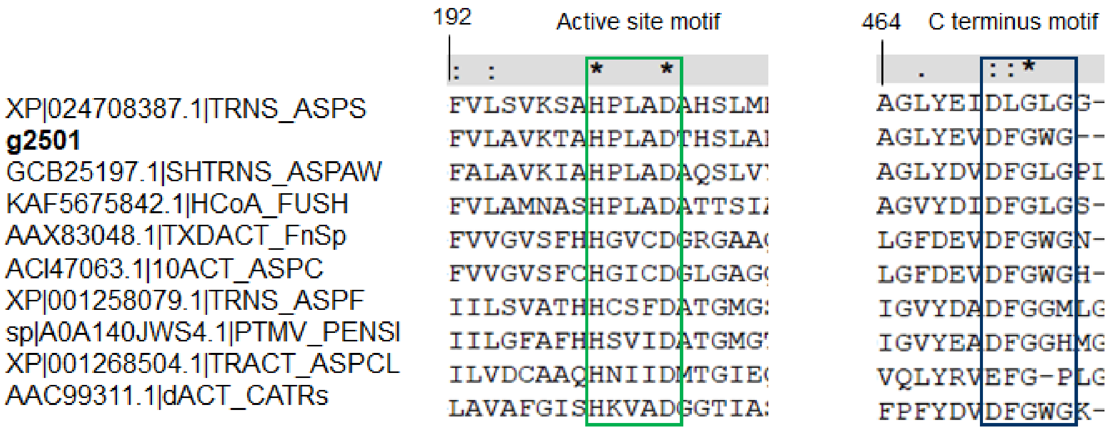

Figure S7. Clustal analysis of the putative g2501 CoA-acetyltransferase (AdoxH). The HXXXD motif (green box) is highly conserved in the BAHD (benzyl alcohol acetyl-, anthocyanin-*O*-hydroxy-cinnamoyl-, anthranilate-*N*-hydroxy-cinnamoyl/benzoyl-, deacetylvindoline acetyltransferase) enzyme superfamily gene and other acyltransferases. DFGWG motif (blue box) is essential for the catalytic activity of this family of enzymes. The sequence numbering is shown according to AdoxH.

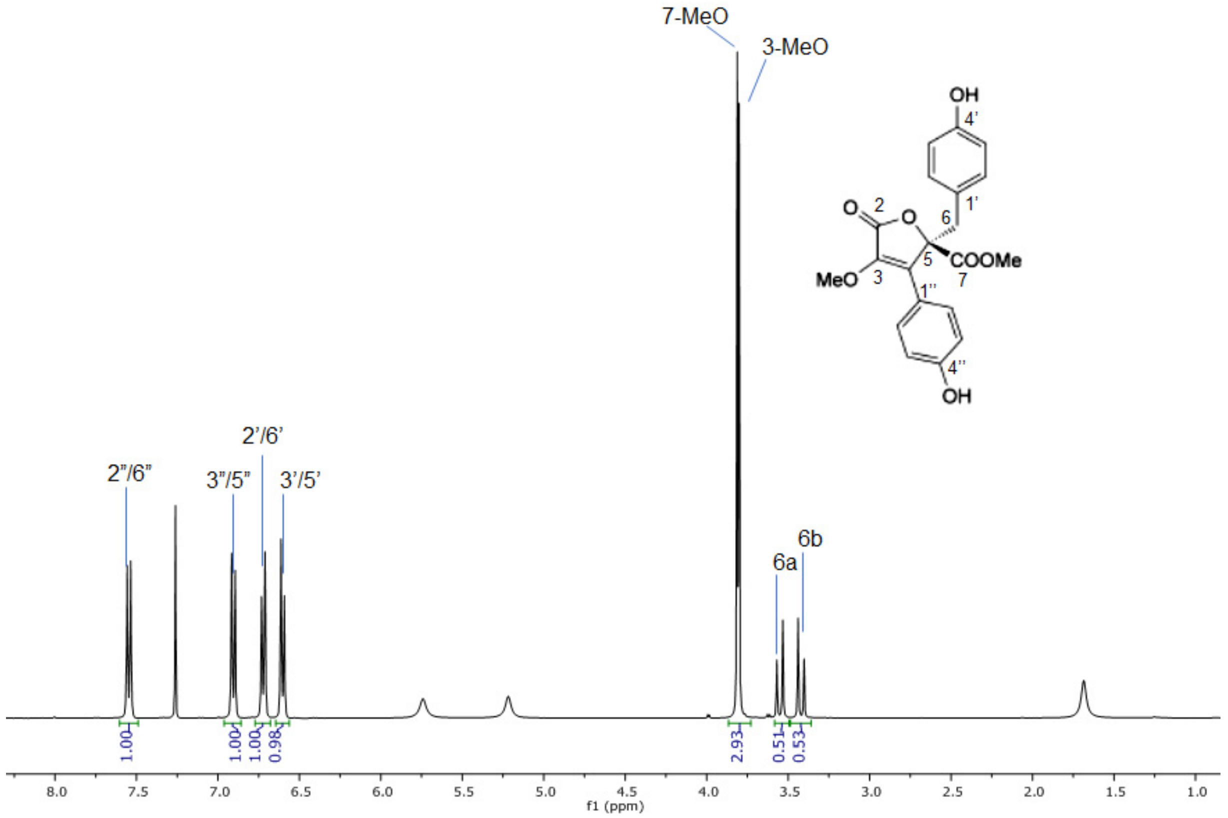

Figure S8. ^{1}H NMR spectrum of 3-*O*-methylbutyrolactone II (**9**) (400 MHz in $CDCl_3$).

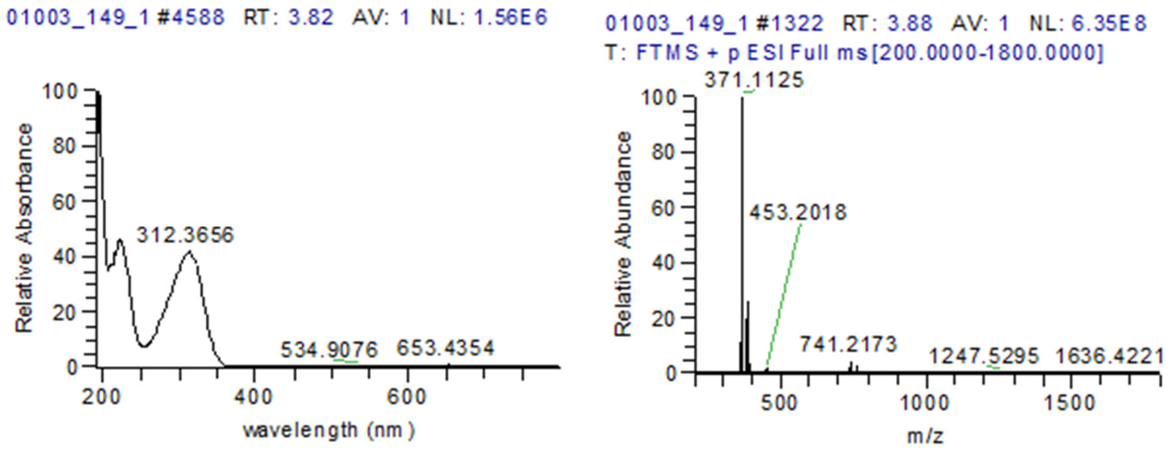

Figure S9. UV (PDA λ 190-500 nm) and HRESIMS (*m/z* 371.1125 $[M+H]^+$, calcd for $C_{20}H_{19}O_7$ 371.1125) spectra of 3-*O*-methylbutyrolactone II (**9**).
