# Supplementary material for: Genome Mining and Molecular Networking-Based Metabolomics of the Marine Facultative Aspergillus sp. MEXU 27854
Source: Molecules. 2021 Sep 3;26(17):5362. doi: 10.3390/molecules26175362 (PMC8433890; doi:10.3390/molecules26175362)
Supplement: Supplementary file 1 [file molecules-26-05362-s001.zip › SI Tables_Aspergillus sp MEXU 27854_Final.pdf]

SUPPORTING INFORMATION TABLES

Table S1. Data of *Aspergillus* spp. genomes in the NCBI.

Organism	Genomes	Assembly Level [a]	GenBank Accession No.*	Size (Mbp) [a]
A. aculeatinus	2	Scaffold	GCF_003184765.1, GCA_003184765.1	36.47
A. aculeatus	1	Scaffold	GCF_001890905.1, GCA_001890905.1	35.42
A. aff. floccosus	1	Scaffold	GCA_001931935.1	31.59
A. alliaceus	2	Scaffold	GCF_009176365.1, GCA_009176365.1	40.17
A. amoenus	1	Scaffold	GCA_009812435.1	35.48
A. arachidicola	2	Scaffold	GCA_009193545.1	39.76
A. avenaceus	1	Scaffold	GCA_009193465.1	33.77
A. awamori	1	Contig	GCA_003850985.1	38.60
A. awamori var. *piceus*	1	Scaffold	GCA_001599415.1	36.06
A. bertholletiae	1	Scaffold	GCA_009193595.1	37.47
A. bombycis	1	Contig	GCF_001792695.1, GCA_001792695.1	37.47
A. brasiliensis	1	Scaffold	GCA_001889945.1	35.80
A. brunneoviolaceus	1	Scaffold	GCF_003184695.1, GCA_003184695.1	37.48
A. burnettii	1	Scaffold	GCA_013421405.2	41.00
A. caelatus	1	Scaffold	GCF_009193585.1, GCA_009193585.1	40.02
A. calidoustus	1	Scaffold	GCA_001511075.1	41.10
A. campestris	1	Contig	GCF_002847485.1, GCA_002847485.1	28.26
A. candidus	1	Scaffold	GCF_002847045.1, GCA_002847045.1	27.32
A. carbonarius	1	Scaffold	GCA_001990825.1	36.15
A. cejpii	1	Contig	GCA_004769165.1	23.89
A. chevalieri	2	Complete genome	GCA_016861735.1	29.70
A. clavatus	1	Scaffold	GCF_000002715.2	27.86
A. coremiiformis	1	Scaffold	GCA_009193565.1	30.13
A. costaricaensis	1	Scaffold	GCF_003184835.1, GCA_003184835.1	36.95
A. cristatus	2	Contig	GCA_001693355.1	27.70
A. elegans	1	Contig	GCA_016097275.1	36.78
A. ellipticus	1	Scaffold	GCA_003184645.1	42.87
A. eucalypticola	1	Contig	GCF_003184535.1, GCA_003184535.1	34.79
A. felis	3	Complete genome	GCA_016413765.1	33.03
A. fijiensis	1	Scaffold	GCF_003184825.1, GCA_003184825.1	36.51
A. fischeri	5	Scaffold	GCF_000149645.2	31.67
A. flavus	108	Complete genome	GCA_014784225.1, GCA_009856665.1, GCA_009017415.1	37.81, 37.53, 37.75
A. floridensis	1	Scaffold	GCA_012184565.1	35.39
A. fumigatiaffinis	4	Scaffold	GCA_012656285.1	33.47
A. fumigatus	23	Chromosome	GCF_000002655.1	29.38
A. fumigatus var. *RP-2014*	1	Scaffold	GCA_000731615.1	27.36
A. fumigatus var. *fumigatus*	1	Scaffold	GCA_001599455.1	28.39
A. glaucus	1	Scaffold	GCF_001890805.1, GCA_001890805.1	27.99
A. hancockii	1	Scaffold	GCA_001696595.2	36.99
A. heteromorphus	1	Contig	GCF_003184545.1, GCA_003184545.1	35.61
A. hiratsukae	2	Scaffold	GCA_014281905.1	30.07
A. homomorphus	1	Scaffold	GCF_003184865.1, GCA_003184865.1	34.05
A. ibericus	1	Scaffold	GCF_003184845.1, GCA_003184845.1	33.44
A. incahuasiensis	1	Scaffold	GCA_003719405.1	31.01
A. indologenus	1	Scaffold	GCA_003184685.1	38.59
A. japonicus	2	Scaffold	GCF_003184785.1, GCA_003184785.1	36.10
A. latus	7	Scaffold	GCA_013306195.1	63.57
A. lentulus	13	Scaffold	GCF_010724455.1, GCA_010724455.1	30.23
A. leporis	1	Scaffold	GCA_009176345.1	39.43
A. luchuensis	13	Complete genome	GCF_016861625.1, GCA_016861625.1	37.26
A. melleus	1	Contig	GCA_016097325.1	38.85
A. minisclerotigenes	1	Scaffold	GCA_009176455.1	37.06

A. mulundensis	1	Scaffold	GCF_003369625.1, GCA_003369625.1	45.34
A. nanangensis	1	Scaffold	GCA_015844325.1	36.25
A. neoellipticus	1	Scaffold	GCA_003116565.1	27.52
A. neoniger	1	Scaffold	GCF_003184625.1, GCA_003184625.1	35.42
A. nidulans	4	Chromosome	GCA_000011425.1	29.83
A. niger	22	Scaffold	GCF_000002855.3	33.98
A. nomiae	2	Contig	GCF_001204775.2, GCA_001204775.2	36.14
A. novofumigatus	1	Contig	GCF_002847465.1, GCA_002847465.1	32.44
A. novoparasiticus	1	Scaffold	GCA_009176405.1	40.86
A. ochraceoroseus	2	Scaffold	GCA_004849945.1	37.02
A. oerlinghausenensis	1	Scaffold	GCA_014250555.1	32.32
A. olivimuriae	1	Scaffold	GCA_003719415.1	30.09
A. oryzae	99	Chromosome	GCA_018140735.1, GCA_018140755.1, GCA_018140865.1, GCF_000184455.2	38.12, 38.65, 37.79, 37.88
A. parasiticus	10	Chromosome	GCA_012897115.1	37.05
**Total**	374			

[a] Data from mayor assembly level.

Table S2. Total predicted genes in the *Aspergillus* sp. MEXU 27854 genome and GO terms *(attached in separate file)*.

Table S3. KEGG pathway classification in the *Aspergillus* sp. MEXU 27854 genome *(attached in separate file)*.

Table S4. Deduced functions of open reading frames (ORFs) in the *adox* BGC.

Name	GB accession No.	Amino Acids	Closest GenBank Homolog	Identity (%)	Similarity (%)
g2501	MZ503791	537	Transferase family protein (*A. steynii* IBT 23096) XP_024708387.1	300/543 (55)	378/543 (69)
			Spermidine hydroxycinnamoyl transferase (*A. awamori*) GCB25197.1	282/549 (51)	357/549 (65)
			Hydroxycinnamoyl-CoA shikimate quinate Hydroxycinnamoyltransferase (*F. heterosporum*) KAF5675842.1	242/531 (46)	347/531 (65)
g7012	MZ503792	426	MAK1-like monooxygenase (*A. flavus*) RAQ69429.1	339/420 (81)	377/420 (89)
			Notoamide E oxidase notB (*Aspergillus* sp. MF297-2) E1ACP7.2	124/408 (30)	192/408 (47)
g7440		490	Cytochrome P450 (*A. leporis*) KAB8067274	378/490 (77)	427/490 (87)
			Isotrichodermin C-15 hydroxylase (*A. awamori*) GCB25848.1	361/490 (74)	414/490 (84)
			Cytochrome P450 monooxygenase notG (*A. versicolor*) L7WME9	129/453 (28)	205/453 (45)
g7441	MZ503793	155	Sterol glucosyltransferase (*F. mundagurra*) KAF5708001	59/138 (43)	84/138 (60)
			Sterol glucosyltransferase (*F. mexicanum*) KAF5548227.1	55/138 (40)	83/138 (60)
7742		261	Cytochrome P450 (*A. brunneoviolaceus*) CBS 621.78	131/299 (44)	180/299 (60)
			Cytochrome P450 *(P. italicum*) KGO77172.1	132/298 (44)	179/298 (60)
g7443		385	Aromatic prenyltransferase, DMATS type (*P. expansum*) XP_016600812	115/371 (31)	187/371 (50)

			adxC (*A. aculeatus* ATCC 16872) XP_020052775	118/365 (32)	168/365 (46)
			Deoxybrevianamide E synthase notF (*Aspergillus* sp. MF297-2) E0Y3X1.1	169/391 (43)	237/391 (60)
g7444		248	Cytochrome P450 (*A. tubingensis*) XP_035358893	98/255 (38)	134/255 (52)
			adxA (*A. aculeatus* ATCC 16872) XP_020052773	1042/2070 (50)	1370/2070 (66)
g7445		2008	Non-ribosomal Peptide Synthetase 10 (*P. subrubescens)* OKP06700	810/2079 (39)	1177/2079 (56)
			Non-ribisomal peptide synthase note (*A. versicolor* L7WU80)	469/1975 (24)	739/1975 (37)
g7446		455	Conserved hypothetical protein (*A. terreus NIH2624*) XP_001213737	374/455 (82)	394/455 (86)
g7447		252	DASH complex subunit Duo1-domain-containing protein (*A. coremiiformis*) KAE8353308	192/244 (79)	212/244 (86)
g7448		191	UPF0132 membrane protein AF_0105 (*A. awamori*) GCB25128	157/194 (81)	172/194 (88)
g7449		915	Cell division control protein (Cdc15) (*A. flavus*) RAQ53306	772/923 (84)	824/923 (89)
g7450		2343	Ccr4-Not transcription complex subunit (*A. sclerotioniger* CBS 115572) XP_025467619	2114/2344 (90)	2230/2344 (95)
g7531	MZ503794	295	methylglyoxal reductase (NADPH-dependent) gre2 (*A. burnettii*) KAF5862445.1	282/333 (85)	314/333 (94)
			adxB (*A. aculeatus* ATCC 16872) XP_020052774	76/339 (22)	135/339 (39)

Table S5. ^{1}H NMR data of 3-*O*-methylbutyrolactone II (**9**).

Position	**3-*O*-methylbutyrolactone II (9)**	
	**δ_H, mult. (*J* in Hz)** [a]	**δ_H, mult. (*J* in Hz)** [b,c]
6a	3.55 (d, *J*=14.7)	3.49 (d, *J*=14.4),
6b	3.42 (d, *J*=14.7)	3.43 (d, *J*=14.4)
3-MeO	3.80 (s)	3.73 (s)
7-MeO	3.81 (s)	3.80 (s)
2'/6'	6.72 (d, *J*=8.4)	6.68 (d, *J*=8.4)
3'/5'	6.60 (d, *J*=8.4)	6.61 (d, *J*=8.4)
2"/6"	7.55 (d, *J*=8.8)	7.54 (d, *J*=8.4)
3"/6"	6.90 (d, *J*=8.8)	6.97 (d, *J*=8.4)

[a] 400 MHz, in $CDCl_3$.
[b] 600 MHz in acetone-d_6.
[c] Chen, M. et al. (**2015**) *Chem. Biodiversity*, 12, 1398-1406.
